# The pathogenic Yersiniae—advances in the understanding of physiology and virulence

**DOI:** 10.3389/fcimb.2013.00051

**Published:** 2013-09-12

**Authors:** Matthew S. Francis

**Affiliations:** Department of Molecular Biology and Umeå Centre for Microbial Research, Umeå UniversityUmeå, Sweden

**Keywords:** *Yersinia*, cellular physiology, metabolism, pathogenicity, host–pathogen interactions, immunity, host responses

Of the ~16 *Yersinia* species, only *Y. pestis*, *Y. pseudotuberculosis*, and *Y. enterocolitica* are pathogenic to humans (Koornhof et al., 1999; Smego et al., 1999). The zoonotic obligate pathogen *Y. pestis* is the causal agent of plague, a systemic disease that is usually fatal if left untreated. Free-living *Y. enterocolitica* and *Y. pseudotuberculosis* are the agents of yersiniosis, a rarely systemic gastrointestinal disease. At the forefront of *Yersinia* research are studies of host cell contact, protein secretion, pathogenesis, immunity and the host response, nutrient sensing and sequestration and the control of gene expression. In this special research topic on the pathogenic Yersiniae is a compilation of reviews and research articles that highlight current knowledge and new developments in these areas of *Yersinia* pathophysiology.

## Bacteria–−host cell contact

*Yersinia* is armored with diverse membrane anchored surface adhesins that each contribute to pathogen–host interactions. Mikula et al. (2013) explore the structure and virulence function of the most prominent of these adhesins. They describe salient roles of select adhesins in intestinal pathogenesis by enteric *Yersinia* and give insight on how altered adhesive potential through specific gene loss or gain may have contributed to *Y. pestis* lifestyle changes. Complementing this is Kolodziejek et al. (2012) that dissects specific physical properties and functional contributions of the esoteric Ail adhesin to *Y. pestis* infections.

## Protein secretion

The Ysc-Yop type III secretion system (T3SS) is encoded on a virulence plasmid common to all human pathogenic *Yersinia* (Cornelis et al., 1998). This injectisome is believed to provide a conduit through which Yop effector toxins can be delivered from the bacterial cytoplasm into the eukaryotic cell cytosol in one step or two (Edgren et al., 2012). Dewoody et al. (2013) provide insight into the regulatory mechanisms controlling injectisome assembly and the hierarchal coordination of substrate secretion. One important regulatory mechanism is the YopN secretion plug, and this is the focus of a study by Joseph and Plano (2013) that demonstrate a stretch of sequence within YopN - corresponding to a site for chaperone binding - is also required for environmental control of Ysc-Yop secretion.

While *Yersinia* pathogenicity is correlated mostly to the plasmid encoded Ysc-Yop T3SS, von Tils et al. (2012) discuss the impact of type II secretion systems (T2SS)—found in the genomes of all pathogenic and non-pathogenic *Yersinia* alike—to bacterial survival in the environment and in the host.

## Immunity and pathogensis

Of the six known Ysc-Yop T3SS translocated effector toxins, YopM function remains enigmatic. As a consequence, Uittenbogaard et al. (2012) performed an exhaustive characterization of early host cell responsiveness to YopM of *Y. pestis*. This revealed exciting new molecular pathways potentially targeted by YopM. Early host responsiveness was also the focus of Vagima et al. (2012), which presents a role for bone marrow derived cells in the early sensing of lung infections by *Y. pestis*. Moreover, recognizing that cell death is a critical attribute of host immunity and microbial pathogenicity, Philip and Brodsky (2012) report on how cell death programs serve as both a *Yersinia* virulence strategy via T3SS-translocated YopJ effector function, as well as an elicitor of innate and adaptive immune responses designed to counteract *Yersinia* infections.

Critical in the evolution of *Y. pestis* has been the acquisition of the gene *pla* encoding an omptin-like outer membrane plasminogen activator protease (Pla). Korhonen et al. (2013) discuss this by summarizing features of controlled Pla protease activation, and its subsequent complex repertoire of interactions with host coagulation and fibrinolysis factors.

## Nutrient sensing and sequestration

A growing theme in infection biology research is the link between carbon metabolism and virulence (Koornhof et al., 2009; Rohmer et al., 2011). Heroven et al. (2012) explore this concept in *Yersinia* pathogenicity using global omics-based profiling. They establish that the carbon storage regulator (Csr) system is controlled by the cAMP receptor protein (Crp), and together customize virulence gene expression according to the prevailing nutrient availability during *Yersinia* infections.

A critical nutrient for growth of all life forms is iron. Rakin et al. (2012) describe an array of independent siderophore-mediated iron sequestration systems encoded by *Y. pestis* and the enteric *Yersinia*. They propose that by acquiring multiple alternative endogenous siderophore systems with unique physiological properties, *Yersinia* has gained the capacity to adapt and thrive in diverse environmental niches.

## Gene expression control

Defining roles for small non-coding RNAs (ncRNAs) in regulation of gene expression dominates the infection biology landscape. Schiano and Lathem (2012) provide examples of small ncRNAs and various other post-transcriptional mechanisms in the regulation of virulence gene expression in *Yersinia*. They even propose that subtle sequence and/or regulatory differences found in certain small ncRNAs could account for some of the acquired lifestyle changes of *Y. pestis*.

Conditions that threaten the integrity of the bacterial envelope are collectively termed extracytoplasmic stresses (ECS). Bacteria encode distinct regulatory pathways designed to maintain bacterial envelope integrity when challenged by ECS. Flores-Kim and Darwin (2012) describe how these ECS responsive pathways are also important for the control of *Yersinia* virulence determinants; particularly those embedded in the bacterial envelope such as integral membrane-spanning T3SSs and the well-known surface adhesin invasin. The environmental control of invasin gene expression in *Y. enterocolitica* is further explored by Brzostkowska et al. (2012). They demonstrate that OmpR, a response regulator of the EnvZ/OmpR two-component regulatory system, binds the *inv* promoter to directly negatively influence invasin expression.

## New frontiers

The systems biology era has revolutionized infectious biology research. Yang et al. (2012) illustrate the power of omics-based explorations in dissecting *Yersinia*-host cell interplay. In so doing, they provide a perspective on the future of omics-based research in benefitting our understanding of *Yersinia* cellular physiology and metabolism as well as host cellular responsiveness and immunity.

Finally, manned space exploration demands an evaluation into the effect of such conditions on microbial virulence and infectious disease communicability. Rosenzweig and Chopra (2012) explain that low shear force conditions reduce the virulence capacity of *Y. pestis*. Thus, knowledge of the mechanisms behind these repressive effects could benefit understanding of *Y. pestis* pathogenicity both in space and on earth.

## References

[B1] Brzostkowska M. Raczkowska A. Brzostek K. (2012). OmpR, a response regulator of the two-component signal transduction pathway, influences *inv* gene expression in *Yersinia enterocolitica* O9. Front. Cell. Infect. Microbiol. 2:153 10.3389/fcimb.2012.00153 23264953 PMC3524506

[B2] Cornelis G. R. Boland A. Boyd A. P. Geuijen C. Iriarte M. Neyt C. (1998). The virulence plasmid of *Yersinia*, an antihost genome. Microbiol. Mol. Biol. Rev. 62, 1315–1352 9841674 10.1128/mmbr.62.4.1315-1352.1998 PMC98948

[B3] Dewoody R. S. Merritt P. M. Marketon M. M. (2013). Regulation of the *Yersinia* type III secretion system: traffic control. Front. Cell. Infect. Microbiol. 3:4 10.3389/fcimb.2013.00004 23390616 PMC3565153

[B3a] Edgren T. Forsberg A. Rosqvist R. Wolf-Watz H. (2012). Type III secretion in Yersinia: injectisome or not? PLoS Pathog. 8:e1002669 10.1371/journal.ppat.1002669 22589714 PMC3349746

[B4] Flores-Kim J. Darwin A. J. (2012). Links between type III secretion and extracytoplasmic stress responses in *Yersinia*. Front. Cell. Infect. Microbiol. 2:125 10.3389/fcimb.2012.00125 23087910 PMC3467454

[B5] Heroven A. K. Sest M. Pisano F. Scheb-Wetzel M. Steinmann R. Bohme K. (2012). Crp induces switching of the CsrB and CsrC RNAs in *Yersinia pseudotuberculosis* and links nutritional status to virulence. Front. Cell. Infect. Microbiol. 2:158 10.3389/fcimb.2012.00158 23251905 PMC3523269

[B6] Joseph S. S. Plano G. V. (2013). The SycN/YscB chaperone-binding domain of YopN is required for the calcium-dependent regulation of Yop secretion by *Yersinia pestis*. Front. Cell. Infect. Microbiol. 3:1 10.3389/fcimb.2013.00001 23355975 PMC3553376

[B7] Kolodziejek A. M. Hovde C. J. Minnich S. A. (2012). *Yersinia pestis* Ail: multiple roles of a single protein. Front. Cell. Infect. Microbiol. 2:103 10.3389/fcimb.2012.00103 22919692 PMC3417512

[B8] Koornhof H. J. Smego R. A. Jr. Nicol M. (1999). Yersiniosis. II: the pathogenesis of *Yersinia* infections. Eur. J. Clin. Microbiol. Infect. Dis. 18, 87–112 10.1007/s100960050237 10219574

[B9] Korhonen T. K. Haiko J. Laakkonen L. Jarvinen H. M. Westerlund-Wikstrom B. (2013). Fibrinolytic and coagulative activities of *Yersinia pestis*. Front. Cell. Infect. Microbiol. 3:35 10.3389/fcimb.2013.00035 23898467 PMC3724046

[B10] Mikula K. M. Kolodziejczyk R. Goldman A. (2013). *Yersinia* infection tools-characterization of structure and function of adhesins. Front. Cell. Infect. Microbiol. 2:169 10.3389/fcimb.2012.00169 23316485 PMC3539135

[B11] Philip N. H. Brodsky I. E. (2012). Cell death programs in *Yersinia* immunity and pathogenesis. Front. Cell. Infect. Microbiol. 2:149 10.3389/fcimb.2012.00149 23226685 PMC3510641

[B12] Poncet S. Milohanic E. Maze A. Nait Abdallah J. Ake F. Larribe M. (2009). Correlations between carbon metabolism and virulence in bacteria. Contrib. Microbiol. 16, 88–102 10.1159/000219374 19494580

[B13] Rakin A. Schneider L. Podladchikova O. (2012). Hunger for iron: the alternative siderophore iron scavenging systems in highly virulent *Yersinia*. Front. Cell. Infect. Microbiol. 2:151 10.3389/fcimb.2012.00151 23226687 PMC3510459

[B14] Rohmer L. Hocquet D. Miller S. I. (2011). Are pathogenic bacteria just looking for food? Metabolism and microbial pathogenesis. Trends Microbiol. 19, 341–348 10.1016/j.tim.2011.04.003 21600774 PMC3130110

[B15] Rosenzweig J. A. Chopra A. K. (2012). The effect of low shear force on the virulence potential of *Yersinia pestis*: new aspects that space-like growth conditions and the final frontier can teach us about a formidable pathogen. Front. Cell. Infect. Microbiol. 2:107 10.3389/fcimb.2012.00107 22919696 PMC3417468

[B16] Schiano C. A. Lathem W. W. (2012). Post-transcriptional regulation of gene expression in *Yersinia* species. Front. Cell. Infect. Microbiol. 2:129 10.3389/fcimb.2012.00129 23162797 PMC3493969

[B17] Smego R. A. Frean J. Koornhof H. J. (1999). Yersiniosis I: microbiological and clinicoepidemiological aspects of plague and non-plague *Yersinia* infections. Eur. J. Clin. Microbiol. Infect. Dis. 18, 1–15 10.1007/s100960050219 10192708

[B18] Uittenbogaard A. M. Chelvarajan R. L. Myers-Morales T. Gorman A. A. Brickey W. J. Ye Z. (2012). Toward a molecular pathogenic pathway for *Yersinia pestis* YopM. Front. Cell. Infect. Microbiol. 2:155 10.3389/fcimb.2012.00155 23248776 PMC3518861

[B19] Vagima Y. Levy Y. Gur D. Tidhar A. Aftalion M. Abramovich H. (2012). Early sensing of *Yersinia pestis* airway infection by bone marrow cells. Front. Cell. Infect. Microbiol. 2:143 10.3389/fcimb.2012.00143 23189271 PMC3505838

[B20] von Tils D. Bladel I. Schmidt M. A. Heusipp G. (2012). Type II secretion in *Yersinia*-a secretion system for pathogenicity and environmental fitness. Front. Cell. Infect. Microbiol. 2:160 10.3389/fcimb.2012.00160 23248779 PMC3521999

[B21] Yang R. Du Z. Han Y. Zhou L. Song Y. Zhou D. (2012). Omics strategies for revealing *Yersinia pestis* virulence. Front. Cell. Infect. Microbiol. 2:157 10.3389/fcimb.2012.00157 23248778 PMC3521224

